# Efficacy of non-conventional synthetic DMARDs for patients with rheumatoid arthritis-associated interstitial lung disease: a systematic review and meta-analysis

**DOI:** 10.1136/rmdopen-2023-003487

**Published:** 2023-10-29

**Authors:** Haoming Yuan, Shaoxin Cui, Lin Yang, Jiehan Cui, Xiaoping Wang, Meng Ding, Lu Jin, Yanru Wang, Fei Chang, Hongtao Jin, Jun Ma, Min Shi, Aijing Liu

**Affiliations:** 1 Department of Rheumatology and Immunology, The Second Hospital of Hebei Medical University, Shijiazhuang, Hebei, China; 2 Hebei Research Center for Stem Cell Medical Translational Engineering, Shijiazhuang, Hebei, China; 3 Department of Anatomy, Hebei Medical University, Shijiazhuang City, Hebei, China; 4 Department of Clinical Laboratory, The Second Hospital of Hebei Medical University, Shijiazhuang, Hebei, China; 5 Hebei Key Laboratory of Laboratory Medicine, The Second Hospital of Hebei Medical University, Shijiazhuang, Hebei, China

**Keywords:** Arthritis, Rheumatoid, Pulmonary Fibrosis, Biological Therapy, Antirheumatic Agents, Qualitative research

## Abstract

**Objectives:**

We conducted a systematic review and meta-analysis to determine the efficacy of non-conventional synthetic disease-modifying antirheumatic drug (ncs-DMARD) strategies on patients with rheumatoid arthritis (RA)-associated interstitial lung disease (ILD).

**Methods:**

PubMed, EMBASE, the Cochrane Library and Web of Science were searched for relevant articles from inception to 1 June 2022. The results obtained from the analysis were expressed as mean difference (MD), effect size and 95% CI.

**Results:**

A total of 17 studies, including 1315 patients with RA-ILD, were eligible. The ncs-DMARDs included abatacept, rituximab, tocilizumab, tumour necrosis factor and Janus kinase inhibitors. Compared with the baseline, there were no significant changes in forced vital capacity (FVC), forced expiratory volume in the first second (FEV_1_) and diffusion lung capacity for carbon monoxide (DLCO) values in the pooled data after ncs-DMARD treatment (alone or combined with conventional therapy) (p=0.36 for FVC; p=0.96 for FEV_1_ and p=0.46 for DLCO). Of note, FVC was obviously increased in rituximab subgroup (MD=−4.62, 95% CI −8.90 to −0.33, p=0.03). Also, high-resolution CT non-progression rate and fatality rate due to ILD progression in patients with RA-ILD were 0.792 (95% CI 0.746 to 0.834, p=0.015) and 0.049 (95% CI 0.035 to 0.065, p=0.000), respectively.

**Conclusion:**

ncs-DMARDs alone or combined with conventional therapy might be an optimal and promising treatment for stabilising or improving ILD in patients with RA-ILD.

**PROSPERO registration number:**

CRD42022356816.

**WHAT IS ALREADY KNOWN ON THIS TOPIC**

- Interstitial lung disease (ILD) is the most common extra-articular manifestation of rheumatoid arthritis (RA). For the past of years, non-conventional synthetic disease-modifying antirheumatic drugs (ncs-DMARDs) have been widely used in patients with RA. However, therapeutic impacts of ncs-DMARDs on patients with RA-ILD were controversial in the previous literature.

**WHAT THIS STUDY ADDS**

- In this study, almost all ncs-DMARDs used in RA were comprehensively analysed to explore their effects on the pulmonary changes of patients with RA-ILD. The pooled results showed that the pulmonary conditions of patients after ncs-DMARD treatment were stable, relatively.

**HOW THIS STUDY MIGHT AFFECT RESEARCH, PRACTICE OR POLICY**

- Our results indicated ncs-DMARD therapy favours stable or improved lung disease in patients with RA.

## Introduction

Rheumatoid arthritis (RA) is a systemic autoimmune disease characterised by destructive joint disease and extra-articular manifestation.^1^ Interstitial lung disease (ILD) is the most common extra-articular manifestation, affecting 2–17% of patients with RA based on clinical symptoms and high-resolution CT (HRCT) chest scan findings.^2–4^ The severe ILD caused by RA negatively affects people’s health worldwide.^5^ It has been reported that the death hazard rate in patients with RA-ILD is 2–10 times that of patients with RA without ILD,^6^ making it the second leading cause of premature death in patients with RA after cardiovascular disease. At present, the exact pathogenesis of RA-ILD remains unclear. It was found that the MUC5B promoter variant, elevated interleukin-17A receptor and citrullination in the lung tissue may contribute to the onset and progression of RA-ILD.^7–9^ It is also worth noting that apart from the natural course of RA and infection, disease-modifying antirheumatic drugs (DMARDs) may induce or worsen ILD.^10^ In this situation, making the decision with patients in therapeutic regimens is more complicated.

In recent years, non-conventional synthetic DMARDs (ncs-DMARDs), including biological DMARDs (bDMARDs) and target DMARDs (tDMARDs), have been used for patients with RA with poor response or intolerance to conventional synthetic DMARDs (cs-DMARDs).^11^ bDMARDs generally include interleukin-6 receptor inhibitors, anti-CD20 monoclonal antibody, tumour necrosis factor inhibitors (TNFis) and costimulatory molecular receptor such as CTLA-4Ig, while tDMARDs encompass Janus kinase inhibitors (JAKis), including tofacitinib (JAK 1 and 3 inhibitors) and baratinib (JAK 1 and 2 inhibitors).

Growing evidence highlights that ncs-DMARDs for RA have a good curative effect^12 13^; however, their therapeutic impact on the progression and outcomes of ILD in patients with RA is controversial. Antoniou *et al*^14^ found that patients with RA-ILD had significantly improved exercise tolerance and stabilised lung function after 1 year of therapy with infliximab. On the contrary, Lindsay *et al*^15^ showed that a patient with RA-ILD presented with progressive dyspnoea and severe ground-glass changes in HRCT after 6 weeks of etanercept. Here, we conducted a systematic literature review and meta-analysis to evaluate the impact of ncs-DMARDs on the ILD outcomes in patients with RA to provide therapeutic decisions for ncs-DMARDs in patients with RA-ILD.

## Methods

The present study was registered on PROSPERO (CRD42022356816) and performed based on the Preferred Reporting Items for Systematic Reviews and Meta-analyses guidelines.^16^

### Search strategy

PubMed, the Cochrane Library, EMBASE and Web of Science Databases were searched for relevant literature published from inception to 1 June 2022, using the following search terms: rheumatoid arthritis, interstitial lung disease and ncs-DMARDs approved for treatment of RA (online supplemental appendix 1).

10.1136/rmdopen-2023-003487.supp1 Supplementary data

### Study selection

Two investigators (HY/SC) independently scanned the titles and abstracts of all retrieved articles to select matched studies. Any researcher disagreements were resolved through discussion, and a third person (LY) determined the final result. The inclusion criteria were as follows: (1) the study population included patients diagnosed with adult RA-ILD, without consideration of gender, race and pattern of ILD of patients; (2) the therapeutic regimen of patients included the application of ncs-DMARDs alone (at least one dose) or in combination with others; (3) clinical trials as well as cohort and case–control studies. Exclusion criteria were as follows: (1) non-English literature; (2) repeatedly published data; (3) literature with incomplete data or lacking target indicators; (4) review articles, letters, conference proceedings, editorials and case reports.

### Data extraction and outcome measures

Two reviewers (HY/SC) independently extracted the following information by using a data extraction form: first author, published year, country, research type, sample size (female/male), mean age, duration of RA-ILD, follow-up time, pattern of ILD, other airway diseases, types of ncs-DMARDs, adverse events (AEs) and indicators. In the case of unavailability of detailed information, the data were obtained by contacting the original author through email. The Cochrane risk of bias assessment tool and Newcastle–Ottawa Scale (NOS) were used to evaluate the bias risk of randomised controlled trials (RCTs) and non-RCT studies.^17^ The certainty of evidence was assessed using the Grading of Recommendations, Assessment, Development, and Evaluation (GRADE) framework classified with high, moderate, low or very low grade.^18^ The primary outcomes were forced vital capacity (FVC) and diffusion lung capacity for carbon monoxide (DLCO). The second outcomes were forced expiratory volume in the first second (FEV_1_), non-progression rate of HRCT and fatality rate. The non-progression rate of HRCT was defined as the percentage of stable or improved images in patients with RA-ILD who underwent HRCT. The fatality rate was calculated as the percentage of patients with RA-ILD who died due to ILD progression.

### Statistical analysis

The extracted data, including FVC, FEV_1_ and DLCO, were analysed using Revman V.5.4 software (Cochrane Collaboration). Single-group results (eg, non-progression rate of HRCT and fatality rate) were pooled and analysed by using STATA V.16.0. For continuous outcomes, pooled outcomes were presented as a mean difference (MD) and 95% CI for analysis, while for single-group rates, effect size and 95% CI were used for analysis. The I^2^ value was used to evaluate heterogeneity. Generally, we used the fixed-effects model to analyse substantial homogeneous trials (I^2^≤50%, p>0.1). When statistical heterogeneity existed (I^2^>50%, p<0.1), we used a random-effects model followed by sensitivity analyses and subgroup analyses, which were carried out by gradually removing studies and performed according to types of drugs. STATA V.16.0 software and Egger’s test were used to evaluate publication bias for studies involving FVC and DLCO.

## Results

### Study selection

**Figure 1:**
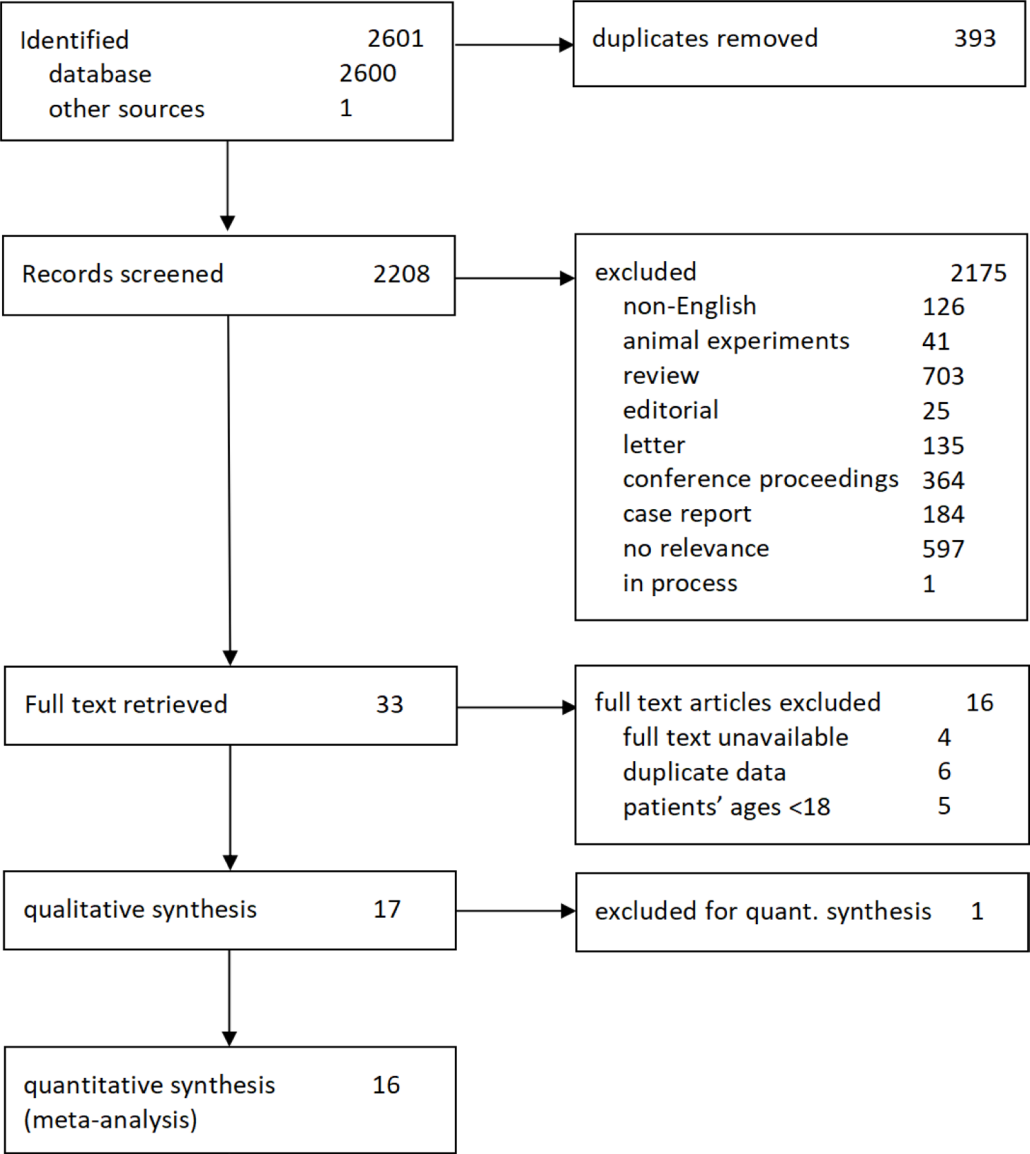
Flow chart of included studies and excluded reasons.

According to the above search strategy, 2601 articles were initially retrieved from five databases, and 2208 were acquired after the removal of duplicates. Next, the titles and abstracts of articles were screened for potential eligibility, and 33 were considered for full-text review, which met the inclusion criteria. Finally, 17 studies, including 16 quantitative studies, were identified. All the included patients met the 1987 American College of Rheumatology (ACR) or the 2010 ACR/European Alliance of Associations for Rheumatology classification criteria of RA. Details of the study screening are shown in figure 1.

### Study characteristics

A total of 17 clinical studies with 1315 adult patients with RA-ILD were included, all of which were self-controlled studies. The studies were conducted in seven different countries, more precisely four in Spain,^19–22^ five in Italy,^23–27^ one in Greece,^28^ four in Britain,^29–32^ one in South Korea,^33^ one in Japan^34^ and one in the USA.^35^ The age range of patients was 49–83 years, and the follow-up time was approximately 6–70 months. There were three studies with abatacept (ABA) treatment,^20 23 27^ eight with rituximab (RTX),^19 21 22 25 30–32 35^ one with tocilizumab (TCZ),^26^ four with TNFis,^28–30 33^ two with JAKis^24 27^ and one without detailed information of bDMARDs.^34^ The concomitant cs-DMARDs involved methotrexate (MTX), azathioprine (AZA), mycophenolate mofetil (MMF), leflunomide (LEF), tacrolimus (TAC), immunoglobulins, sulfasalazine (SSZ) and hydroxychloroquine (HCQ). Of all the 1315 patients, 231 were classified as usual interstitial pneumonia (UIP) by HRCT, 200 non-specific interstitial pneumonia (NSIP), 102 as others, including organising pneumonia, cryptogenic organising pneumonia, mixed patterns, etc. Also, the ILD radiological patterns of the remaining 782 were unknown. Detailed information is shown in table 1.

**Table 1:** Basic characteristics of the included studies

References	Year	Country	Sample size (female/male)	Age (mean±SD or median, IQR) (years)	Duration of RA disease (mean±SD or median, IQR)	Duration of ILD disease (mean±SD or median, IQR)	Follow-up times (mean±SD or median, IQR)	ILD radiological patterns (n)	Other airway disease*, n (%)	Adverse events (n)	Types of ncs- DMARD treatment	Indicators included†	Literature quality evaluation	Score
Atienza-Mateo *et al*^19^	2020	Spain	5 (NA)	NA	NA	NA	6.0 (6.0) months	NA	NA	NA	RTX	①	NOS	5
Cassone *et al*^23^	2020	Italy	44 (32/12)	65.0 (11)	89.0 (142.0) months	20.0 (58.0) months	26.5 (38.0) months	UIP (19); NSIP (22); others‡ (3)	10 (22.7)	Non-respiratory infection (1)	ABA	①③④	NOS	8
d'Alessandro *et al*^24^	2020	Italy	4 (NA)	NA	NA	NA	6.0 (6.0) months	UIP (1); NSIP (2); others‡ (1)	NA	None	JAKis	FEV_1_/FVC	NOS	6
Detorakis *et al*^28^	2016	Greece	42 (27/15)	60.1±7.9	8.9±3.4 years	NA	12.0 (12.0) months	UIP (22); NSIP (12); others‡ (8)	NA	Respiratory infections (3)	TNFis	①②	NOS	8
Dixon *et al*^29^	2010	Britain	299 (57/242)	63.0±10.0	12.0 (7.0–20.0) years	NA	3.8 (2.0–4.7) years	NA	73 (31.9)	NA	TNFis	⑤	NOS	8
Druce *et al*^30^	2017	Britain	352 (198/154)	62.8±10.5	11.1±9.7 years	NA	801.3 (NA) person-years	NA	98 (27.8)	NA	RTX, TNFis	⑤	NOS	8
Duarte *et al*^31^	2019	Britain	26 (NA)	NA	NA	NA	23.3 (6.0–36.0) months	UIP (4); NSIP (10); others‡ (3)	NA	NA	RTX	①④	NOS	7
Fernández-Diaz *et al*^20^	2020	Spain	263 (150/113)	64.6±10.0	9.7±8.7 years	1.0 (0.3–3.4) years	12.0 (6.0–36.0) months	UIP (106); NSIP (84); others‡ (73)	NA	Respiratory infections (25); other infections (3); infusion reaction (1)	ABA	①③④⑤	NOS	8
Fui *et al*^25^	2019	Italy	14 (NA)	62.6±3.2	NA	NA	12.0 (12.0) months	UIP (9); NSIP (3); others‡ (2)	NA	Non-respiratory infection (1); hypogammaglobulinaemia (1)	RTX	①②③	NOS	7
Koo *et al*^33^	2015	Korea	24 (21/3)	68.5±14.8	107.8±51.1 months	NA	19.0 (2.0–58.0) months	UIP (5); NSIP(NA); others‡ (NA)	1 (4.2)	NA	TNFis	⑤	NOS	7
Kurata *et al*^34^	2019	Japan	23 (NA)	NA	NA	NA	70.9±73.4 weeks	NA	6 (26.1)	NA	bDMARDs	④	NOS	8
Manfredi *et al*^26^	2019	Italy	28 (18/10)	64.0 (15.0)	11.5 (13.0) years	12.0 (34.0) months	30.0 (44.0) months	UIP (14); NSIP (13); others‡ (1)	NA	NA	TCZ	①③④	NOS	8
Matteson *et al*^35^	2012	USA	10 (6/4)	64.7±9.3	13.8±10.9 years	3.2±1.9 years	48.0 (48.0) weeks	UIP (4); NSIP (6)	NA	NA	RTX	①③④	NOS	7
Mena-Vázquez *et al*^21^	2022	Spain	19 (13/6)	67.7±9.7	151.0 (8.0–240.4) months	82.2 (37.4–120.1) months	45.3 (22.2–79.9) months	UIP (14); NSIP (5)	NA	Respiratory infections (13); other infections (5)	RTX	①②③④⑤	NOS	9
Narváez *et al*^22^	2020	Spain	31 (18/13)	61.0±12.0	48.0 (19.0–116.0) months	21.0 (9.0–38.0) months	24.0 (24.0) months	UIP (13); NSIP (10); others‡ (8)	NA	Respiratory and other infections (10); hypogammaglobulinaemia (9)	RTX	①③④	NOS	7
Tardella *et al*^27^	2022	Italy	75 (52/23)	59.5±7.7	7.5±3.2 years	NA	18.0 (18.0) months	NA	NA	NA	ABA, JAKis	①③④	NOS	7
Yusof *et al*^32^	2017	Britain	56 (36/30)	64.0 (59.0–72.0)	10.0 (7.0–13.0) years	5.0 (3.0–7.0) years	195 (NA) person-years	UIP (20); NSIP (33); others‡ (3)	NA	Respiratory infections (11); other infections (4); thromboembolism (2); acute coronary syndrome (2); malignancy (3); other hospitalisation§ (44)	RTX	①③④⑤	NOS	8

*Other airway diseases including chronic obstructive pulmonary diseases, asthma and tuberculosis.

†① FVC; ② FEV_1_; ③ DLCO; ④ changes of HRCT; ⑤ fatality rate.

‡Others including organising pneumonia, cryptogenic organising pneumonia, pleuroparenchymal fibroelastosis, bronchiolitis obliterans, lymphoid interstitial pneumonia, hypersensitivity pneumonitis, mixed patterns, etc.

§Other hospitalisation including orthopaedics surgery (elective), lung flare, arthritis flare, gastrointestinal surgery, palliative care and seizure.

ABA, abatacept; bDMARDs, biological disease-modifying antirheumatic drugs; DLCO, diffusion lung capacity for carbon monoxide; FEV_1_, forced expiratory volume in the first second; FVC, forced vital capacity; HRCT, high-resolution CT; ILD, interstitial lung disease; JAKis, Janus kinase inhibitors; NA, not applicable; ncs-DMARD, non-conventional synthetic disease-modifying antirheumatic drug; NOS, Newcastle–Ottawa Scale; NSIP, non-specific interstitial pneumonia; RA, rheumatoid arthritis; RTX, rituximab; TCZ, tocilizumab; TNFis, tumour necrosis factor inhibitors; UIP, usual interstitial pneumonia.

### Quality evaluation of the included studies

A total of 17 articles were evaluated using the NOS,^17^ 3 of which were of moderate quality and 14 were of high quality, as shown in online supplemental table 1. The evidence quality of outcomes judged by GRADE^18^ is depicted in online supplemental table 2. Concerning FVC and DLCO, evidence ranged from moderate to very low, and for HRCT non-progression and fatality rates, evidence ranged from moderate to low. We observed FEV_1_ with moderate quality of evidence.

### FVC and FEV_1_

**Figure 2:**
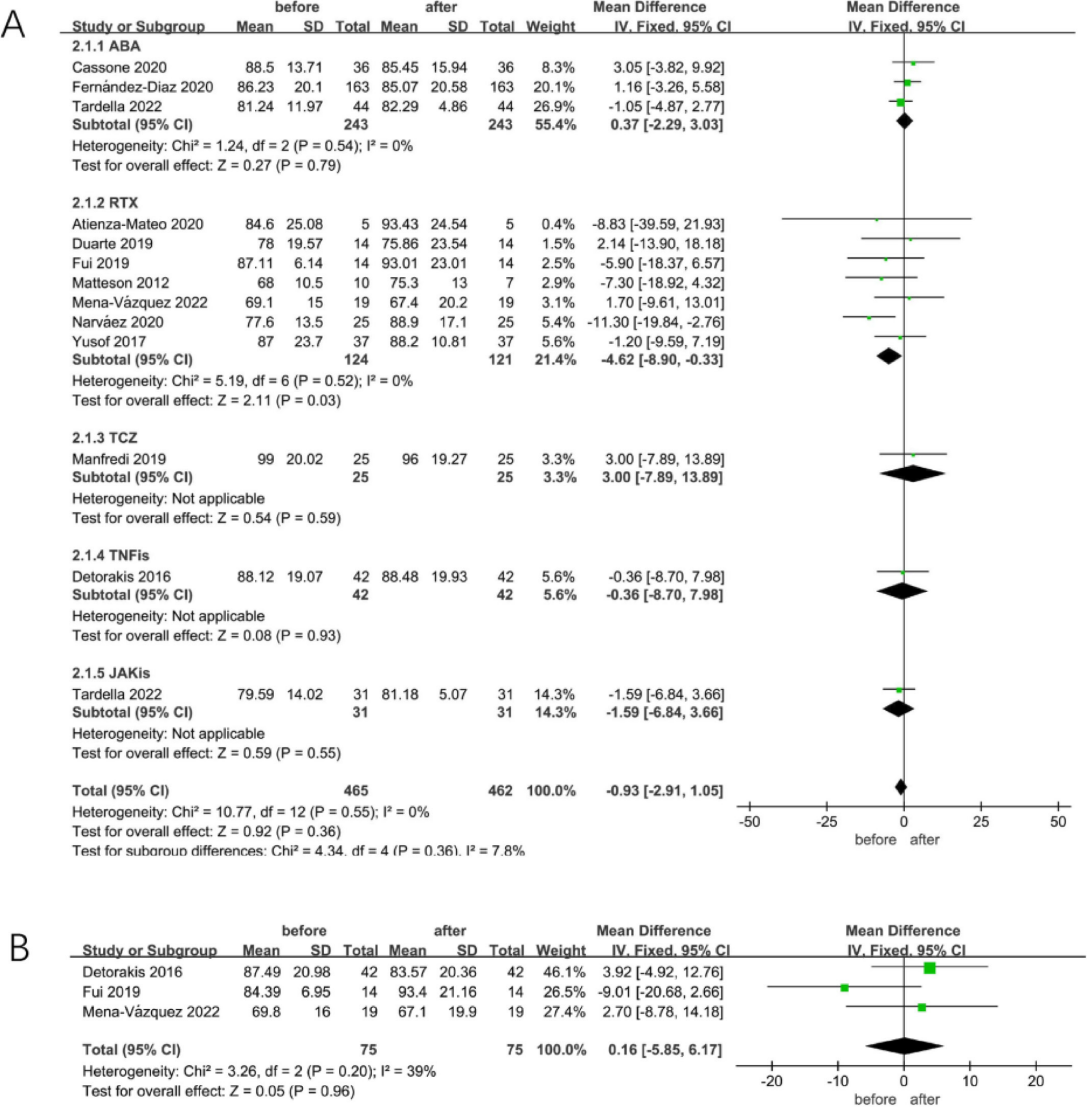
(A) Meta-analysis of self-control studies to assess FVC changes in patients with RA-ILD treated with different types of ncs-DMARDs; (B) meta-analysis of self-control studies to assess FEV_1_ changes in patients with RA-ILD treated with different types of ncs-DMARDs. ABA, abatacept; FEV_1_, forced expiratory volume in the first second; FVC, forced vital capacity; ILD, interstitial lung disease; JAKis, Janus kinase inhibitors; ncs-DMARDs, non-conventional synthetic disease-modifying antirheumatic drugs; RA, rheumatoid arthritis; RTX, rituximab; TCZ, tocilizumab; TNFis, tumour necrosis factor inhibitors.

FVC, one of the crucial components of the pulmonary function test (PFT), is a significant indicator for detecting the resistance of the respiratory tract. In the current study, some patients with RA-ILD presented with abnormalities in FVC. Meta-analysis of 12 studies, including 465 patients with RA-ILD, showed that FVC did not significantly change after being treated with ncs-DMARDs, including ABA, RTX, TNFis, TCZ and JAKis (MD=−0.93, 95% CI −2.91 to 1.05, p=0.36), as shown in figure 2A. A fixed-effects model was used for low heterogeneity for either the pooled or each subgroup (I^2^=0%, p=0.55; figure 2A). The subgroup analysis was performed according to ncs-DMARD types, detecting no significant change in patients with RA-ILD in FVC after receiving ABA but evident amelioration after RTX treatment (MD=0.37, 95% CI −2.29 to 3.03, p=0.79 for ABA; MD=−4.62, 95% CI −8.90 to −0.33, p=0.03 for RTX; figure 2A). Additionally, four patients with RA-ILD who received baricitinib showed a nearly 10% increase in FEV_1_/FVC% after 6 months of follow-up in the research by d’Alessandro *et al.*^24^

FEV_1_ is closely related to FVC, which refers to the volume of exhaled air in the first second of maximum exhalation after maximum deep inspiration. As shown in figure 2B, FEV_1_ changes in three studies, including 75 patients with RA-ILD, were compared before and after ncs-DMARD treatment. The analysis of the fixed-effects model proved that the FEV_1_ remained unchanged in patients with RA-ILD who received ncs-DMARDs (I^2^=39%, p=0.20; MD=0.16, 95% CI −5.85 to 6.17, p=0.96; figure 2B).

### Diffusion lung capacity for carbon monoxide

**Figure 3:**
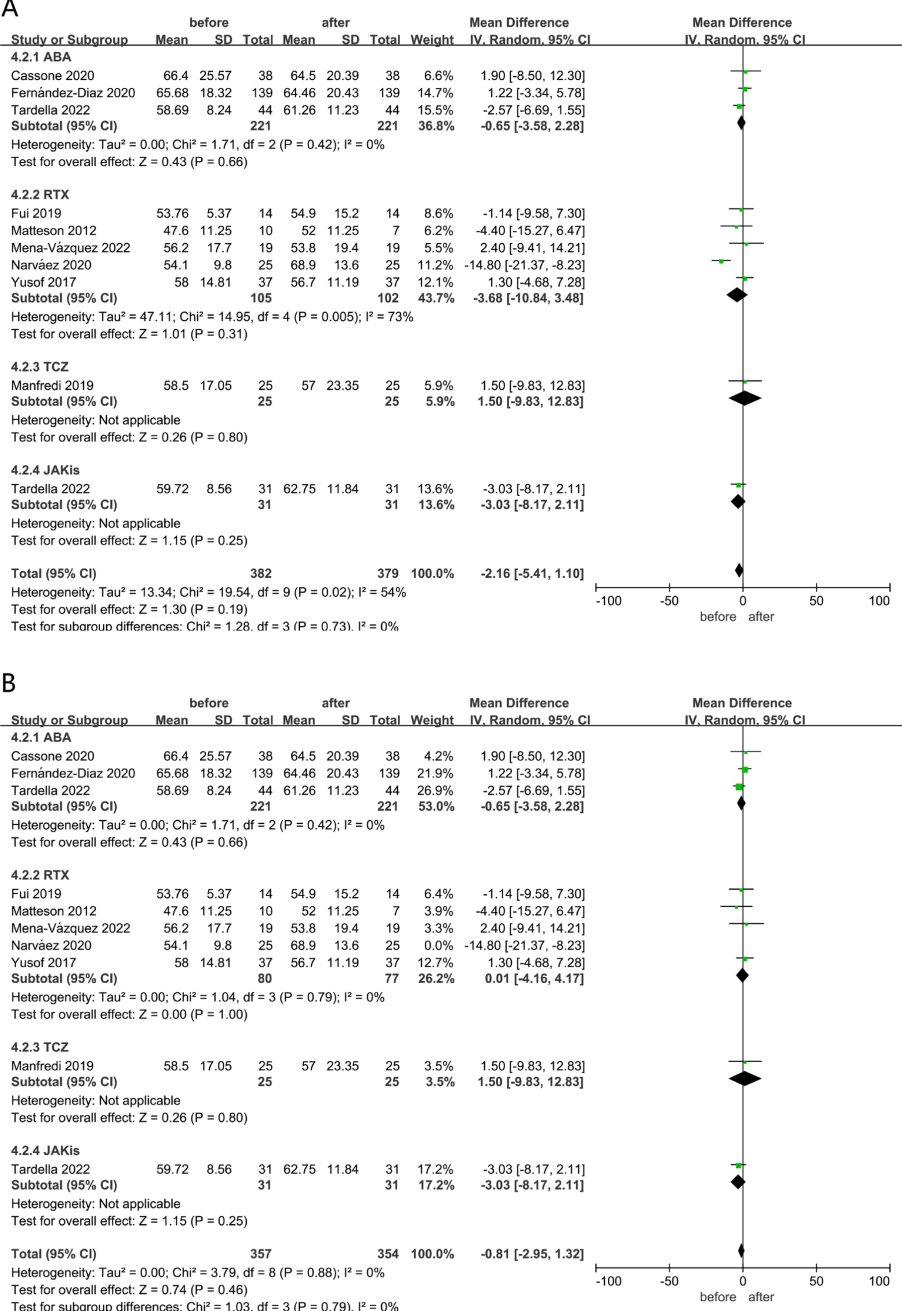
(A) Meta-analysis of self-control studies to assess DLCO changes in patients with RA-ILD treated with different types of ncs-DMARDs; (B) meta-analysis of self-control studies to assess DLCO changes in patients with RA-ILD treated with different types of ncs-DMARDs after sensitivity analysis. ABA, abatacept; DLCO, diffusion lung capacity for carbon monoxide; ILD, interstitial lung disease; JAKis, Janus kinase inhibitors; ncs-DMARDs, non-conventional synthetic disease-modifying antirheumatic drugs; RA, rheumatoid arthritis; RTX, rituximab; TCZ, tocilizumab.

DLCO reflects the diffuse function of the lung. Here, a meta-analysis of nine studies with 313 patients with RA-ILD showed that the result of the pooled DLCO changes was not significant after ncs-DMARDs involving ABA, RTX, TCZ and JAKis (MD=−2.16, 95% CI −5.41 to 1.10, p=0.19), as shown in figure 3A. A random-effects model was used for analysis because of the high heterogeneity (I^2^=54%, p=0.02; figure 3A). Furthermore, we performed sensitivity analysis by sequentially removing studies. As shown in figure 3B, after eliminating Narváez *et al*^22^ in the RTX group with the wide 95% CI, there were no apparent changes in the left of merging results, indicating the outcomes were stable and reliable (MD=−0.81, 95% CI −2.95 to 1.32, p=0.46; figure 3B). The results of subgroup analysis according to the different ncs-DMARDs showed no apparent changes in DLCO in patients with RA-ILD treated with ABA or RTX (I^2^=0%, MD=−0.65, 95% CI −3.58 to 2.28, p=0.66 for ABA; I^2^=0%, MD=0.01, 95% CI −4.16 to 4.17, p=1.00 for RTX; figure 3B). Our results revealed stable lung diffuse function in patients with RA-ILD after ABA or RTX treatment.

### Non-progression rate of HRCT

**Figure 4:**
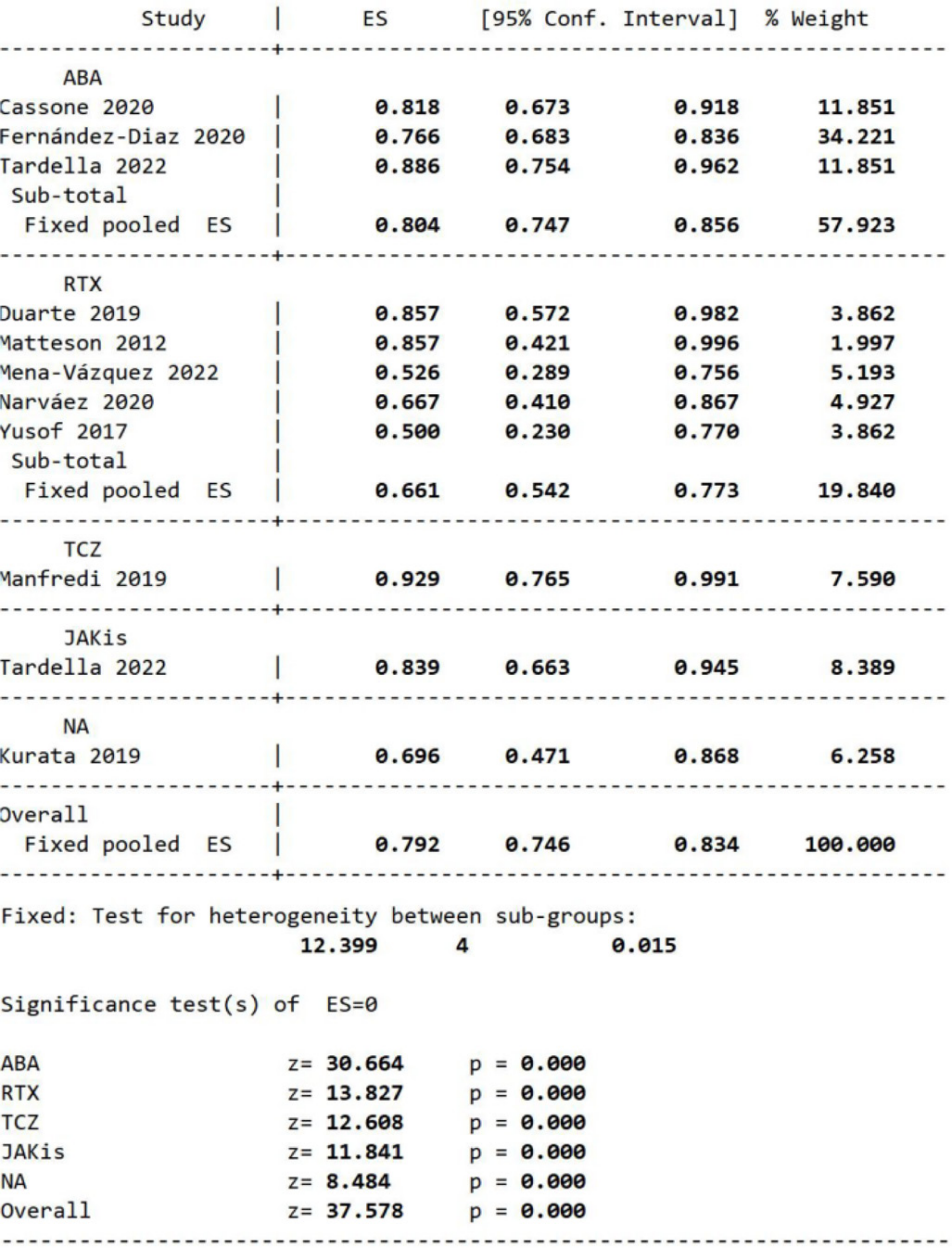
Meta-analysis of self-control studies to assess the single-group non-progression rate of HRCT in patients with RA-ILD treated with ncs-DMARDs. ABA, abatacept; ES, effect size; HRCT, high-resolution CT; ILD, interstitial lung disease; JAKis, Janus kinase inhibitors; NA, not applicable; ncs-DMARDs, non-conventional synthetic disease-modifying antirheumatic drugs; RA, rheumatoid arthritis; RTX, rituximab; TCZ, tocilizumab.

In our meta-analysis, all the HRCT images of patients with RA-ILD were assessed at the baseline and the end of the follow-up time (12–45 months). The assessment was done by an experienced radiologist with a blind approach and identified by another senior radiologist if there were suspicious cases. Our results demonstrated that the HRCT non-progression rate in patients with RA-ILD treated with ncs-DMARDs (ABA, RTX, TCZ, JAKis) was 0.792 (95% CI 0.746 to 0.834, p=0.000 by STATA V.16.0 software; figure 4) in 399 patients from 10 studies, which means the pulmonary radiographical images of majority of the patients with RA-ILD were steady or improved. Next, we used a homogeneous fixed-effects model to analyse the subgroup data based on ncs-DMARD types (I^2^=12.399%; figure 4), finding that the non-progression rate of HRCT in patients treated with ABA was 0.804 (95% CI 0.747 to 0.856, p=0.000), and the rate of RTX was 0.661 (95% CI 0.542 to 0.773, p=0.000; 80.4% vs 66.1%; figure 4).

### Fatality rate

**Figure 5:**
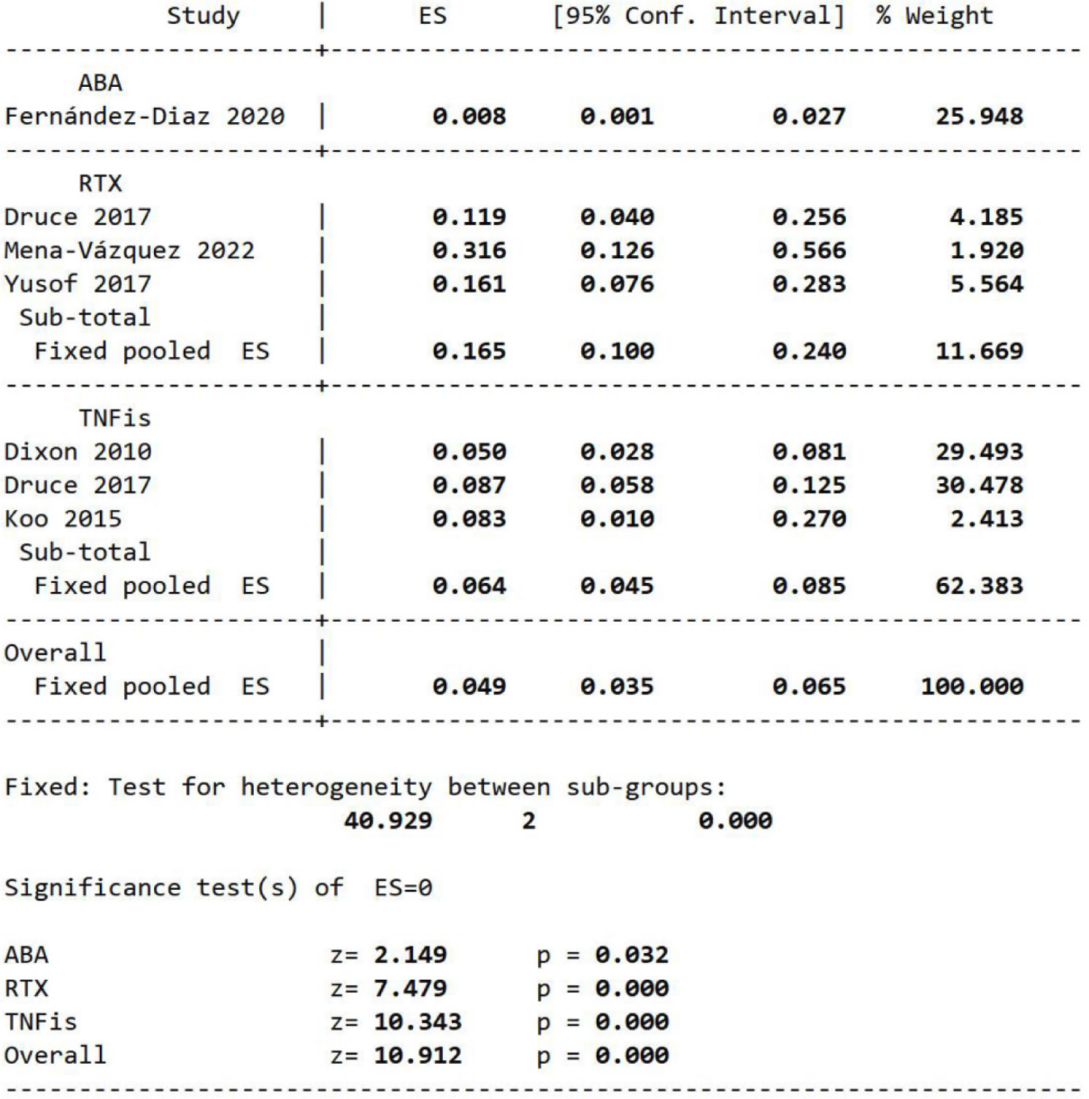
Meta-analysis of self-control studies to assess the single-group case fatality rate of patients due to ILD progression in patients with RA-ILD treated with ncs-DMARDs. ABA, abatacept; ES, effect size; ILD, interstitial lung disease; ncs-DMARDs, non-conventional synthetic disease-modifying antirheumatic drugs; RA, rheumatoid arthritis; RTX, rituximab; TNFis, tumour necrosis factor inhibitors.

Our meta-analysis of single-group rate in five studies involving 749 patients with RA-ILD with ncs-DMARD treatment (ABA, RTX and TNFis) showed that after 3.8 years, the fatality rate due to ILD deterioration was 0.049 (95% CI 0.035 to 0.065, p=0.000; I^2^=40.929%; figure 5) in homogeneous fixed-effects model. Subgroup analysis revealed that the fatality rate of patients with RA-ILD treated with RTX was 0.165 (95% CI 0.100 to 0.240, p=0.000), and the rate of TNFis was 0.064 after 3.8 years (95% CI 0.045 to 0.085, p=0.000; 16.5% vs 6.4%; figure 5).

### Publication bias

**Figure 6:**
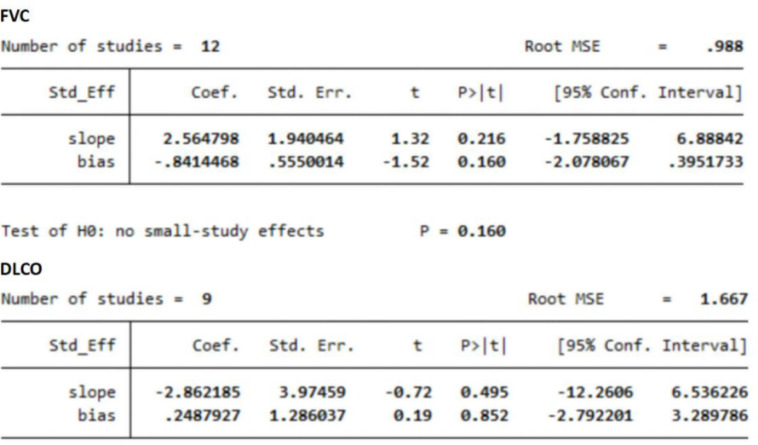
Egger’s test to assess the publication bias of primary outcome. MSE, mean squared error; DLCO, diffusion lung capacity for carbon monoxide; FVC, forced vital capacity.

Regarding FVC and DLCO in patients with RA-ILD, the p values of Egger’s test were all >0.05 (FVC, p=0.160; DLCO, p=0.852; figure 6), indicating no publication bias.

## Discussion

After repeated screening and checking, 17 self-controlled studies encompassing 1315 patients with RA-ILD were included. Despite different types of ncs-DMARDs, our data showed that ncs-DMARD therapeutic regimen might effectively stabilise PFT or HRCT and decrease the fatality rate of patients with RA-ILD owing to progressive ILD.

To explore the impact of ncs-DMARDs on ILD in patients with RA, we also focused on indicators including FVC, FEV_1_, DLCO and HRCT changes. Our results showed that the overall FVC data indicated the stable pulmonary situation of patients after treatment, achieving higher statistical significance after 6–70 months of follow-up in the RTX group, suggesting improving lung function in patients with RA-ILD. In particular, Fernandez-Diaz *et al* showed that FVC of patients with RA-ILD with ABA treatment steadily rose (86.2% to 87.6%) after 12 months of follow-up, which was followed by a slightly downward trend (86.2% to 85.1%) at the end of 22 months.^20^ This was probably due to the progression of the primary disease, which suggests that more aggressive therapeutic drugs should be considered. Regarding FEV_1_, our results detected no statistical significance in patients with RA-ILD while indicating that ncs-DMARD therapy could stabilise patients’ lung function. Unsurprisingly, the FEV_1_, which is closely related to FVC, was not significantly improved in the RTX group because of insufficient data.

In addition, regardless of whether Narváez *et al*’s study was excluded from the sensitivity analysis or not,^22^ DLCO of patients with RA-ILD treated with ABA and RTX was relatively stable, suggesting that patients did not have an apparent deterioration of lung diffuse function. Meanwhile, Tardella *et al*^27^ showed that JAKis combined with steroids might slightly improve the DLCO of the patients (59.7% to 62.8%). Consistent with the above observations, in some case reports, JAKis regimen improved DLCO without pulmonary deterioration or infection in patients with RA-ILD after 8–12 months of follow-up.^36 37^

As shown in figure 4, 11 of 19 studies calculated the non-progression rate of HRCT after treatment with ncs-DMARDs. Additionally, the HRCT pattern of ILD made a difference in the prognosis of RA. One identified UIP as the predominant pattern^38^; however, in our population, the percentages of patients with a UIP pattern versus an NSIP pattern were similar (18% vs 15%, respectively). Nonetheless, with 782 patients with unknown patterns, it is challenging to truly know the predominant radiological pattern of ILD in our pooled population. Of the five studies in this report,^22 23 31 32 35^ radiographical worsening data were recorded in 15 of the 38 patients with UIP patterns. In contrast, only 9 of the 53 patients with NSIP pattern had deterioration of HRCT, indicating the NSIP type may better respond to ncs-DMARDs. Of note, HRCT after RTX treatment was not routinely performed in all patients (if stable), which might decrease the real non-progression rate in the RTX group.

It was reported that the median survival of patients with RA after diagnosis of ILD was 2.6–8.5 years in different US cohort studies, which was significantly shorter than the expected years of patients with RA of the same age and sex, about 16% of whom died from ILD deterioration.^39 40^ For the death of patients with RA-ILD treated with ncs-DMARDs, our meta-analysis revealed that with 3.8-year follow-up, the fatality rate due to ILD deterioration (ILD as the main cause of death) was 6.4% and 16.5%, respectively in TNFis and RTX subgroup. Notably, the pulmonary comorbidities (such as chronic obstructive pulmonary disease or asthma) in patients from the RTX group were more severe (41.9%).^30^ On the contrary, Kelly *et al* found that RTX therapy was more effective than our results suggested (4% vs 16.5%), although it was based on respiratory mortality, including that caused by infection or pulmonary embolism.^41^ These differences were explained by inconsistency in the evaluation criteria of death cause, ILD severity, HRCT patterns and pulmonary comorbidities, all of which might contribute to the relatively high case fatality rate in the RTX group observed in the present study. At the same time, the other differences in sample sizes should not be ignored. In our data, the other AEs including infections (respiratory, n=62; others, n=14),^20–23 25 28 32^ hospitalisation owing to non-infections (n=48),^32^ local infusion reaction (n=1)^20^ and hypogammaglobulinaemia (n=10)^22 25^ were reported during ncs-DMARD treatment. However, there was no exact link between infection and the combined use of ncs-DMARD strategies.

As far as drug details are concerned, RTX and TNFis were entirely applied with either cs-DMARDs (MTX, AZA, MMF, LEF, TAC, SSZ, HCQ) or glucocorticoids, while ABA, TCZ and JAKis were recorded as monotherapy or combined regimen. The overall risk of lung exacerbation attributed to MTX is controversial. Some research reported that MTX treatment led to RA-ILD progression in patients with RA.^27 42^ Nevertheless, in our literature, the majority of studies indicated that MTX did not cause the progression of ILD.^23 28 34^ Similarly, growing studies suggested that MTX in monotherapy or combined with bDMARDs was not associated with an increased risk of RA-ILD in patients, actually it might be equally effective and safe.^2 43 44^ Importantly, the 2021 ACR guidelines for RA treatment conditionally favour MTX for patients with mild and stable airway or parenchymal lung disease, because of the anchor status as a DMARD and lack of alternatives with efficacy and long-term safety.^45^ As glucocorticoids with stable low doses (eg, prednisolone and methylprednisolone) were found to be used in most patients with RA throughout the follow-up, we confirmed that steroid use had no impact on ILD outcomes in our data. It is worth mentioning that antifibrotic drugs (eg, nintedanib and pirfenidone) were not applied in the patients enrolled in our research, providing little therapeutic influence on our final results.

Undoubtedly, increased evidence indicates rheumatoid factor (RF) and/or anti-cyclic citrullinated protein (CCP) seropositivity has been the risk factors for ILD susceptibility.^46 47^ Of the 11 studies in our data, the RF and anti-CCP seropositivity were recorded in 727 of 891 (81%) and 295 of 339 (87%) patients, respectively.^20–23 26–29 32 33 35^ Regarding the relationship between the RF or anti-CCP seropositivity and the effect of the biological treatments on the progression of ILD, four of our included studies indicated that the RF seropositivity was irrelevant to RA-ILD deterioration after ncs-DMARD treatment (ABA, JAKi, TNFi)^23 27 28 34^; inversely, Detorakis *et al* found that serum levels of anti-CCP considerably decreased in the RA-ILD group following TNFi treatment.^28^ More studies with stratified seropositive or seronegative group are needed to provide detailed information.

Nevertheless, the present study has some limitations. First, the included literature was mainly self-controlled studies, where the natural course of the disease might interfere with clinical outcomes. Simultaneously, we should consider the impact of combining regimens on disease progression. Future well-designed RCTs with different ncs-DMARD therapy groups (monotherapy, combination and cs-DMARDs alone) on RA-ILD are needed to further confirm reported findings. Second, the outcomes of certain ncs-DMARDs such as TCZ and JAKis were insufficient due to few eligible studies, and data from relevant clinical trials with large sample sizes are essential. Finally, due to confounding factors, it is necessary to conduct stratified analysis, for example, comorbidities, radiographical patterns and the severity of ILD. A new concept, progressive pulmonary fibrosis (PPF), was proposed in 2022. It was defined by meeting at least two of three criteria (worsening symptoms, radiological progression and physiological progression) occurring in the past year without an alternative explanation other than idiopathic pulmonary fibrosis in patients with ILD.^48^ Hence, it is critical to recognise early PPF state in patients with RA-ILD and timely adjust treatment regimen in order to improve patients’ prognosis.

## Conclusion

Our data suggest that ncs-DMARD therapy might stabilise FVC, FEV_1_ and DLCO values in the pooled data. Of note, there was a significant improvement in FVC changes in the RTX subgroup. In addition, patients’ HRCT non-progression and fatality rates after ncs-DMARD treatment were 79.2% and 4.9%, respectively. Generally speaking, ncs-DMARDs alone or combined with conventional therapy might be an optimal and promising treatment that could stabilise lung function and arrest the progression of ILD in patients with RA-ILD.

## Data Availability

Data are available in a public, open access repository. All data relevant to the study are included in the article or uploaded as supplemental information.

## References

[1] Solomon JJ, Brown KK. Rheumatoid arthritis-associated interstitial lung disease. Open Access Rheumatol 2012;4:21–31. 10.2147/OARRR.S14723 27790009 PMC5045096

[2] Juge P-A, Lee JS, Lau J, et al. Methotrexate and rheumatoid arthritis associated interstitial lung disease. Eur Respir J 2021;57:2000337. 10.1183/13993003.00337-2020 32646919 PMC8212188

[3] Vicente-Rabaneda EF, Atienza-Mateo B, Blanco R, et al. Efficacy and safety of abatacept in interstitial lung disease of rheumatoid arthritis: a systematic literature review. Autoimmun Rev 2021;20:102830. 10.1016/j.autrev.2021.102830 33887489

[4] McDermott GC, Doyle TJ, Sparks JA. Interstitial lung disease throughout the rheumatoid arthritis disease course. Curr Opin Rheumatol 2021;33:284–91. 10.1097/BOR.0000000000000787 33625044 PMC8268047

[5] England BR, Hershberger D. Management issues in rheumatoid arthritis-associated interstitial lung disease. Curr Opin Rheumatol 2020;32:255–63. 10.1097/BOR.0000000000000703 32141954 PMC7331796

[6] Hyldgaard C, Hilberg O, Pedersen AB, et al. A population-based cohort study of rheumatoid arthritis-associated interstitial lung disease: comorbidity and mortality. Ann Rheum Dis 2017;76:1700–6. 10.1136/annrheumdis-2017-211138 28611082

[7] Sparks JA. Towards clinical significance of the MUC5B promoter variant and risk of rheumatoid arthritis-associated interstitial lung disease. Ann Rheum Dis 2021;80:1503–4. 10.1136/annrheumdis-2021-220856 34344705

[8] Zhang J, Wang D, Wang L, et al. Profibrotic effect of IL-17A and elevated IL-17Ra in idiopathic pulmonary fibrosis and rheumatoid arthritis-associated lung disease support a direct role for IL-17A/IL-17Ra in human fibrotic interstitial lung disease. Am J Physiol Lung Cell Mol Physiol 2019;316:L487–97. 10.1152/ajplung.00301.2018 30604628

[9] Paulin F, Doyle TJ, Fletcher EA, et al. Rheumatoid arthritis-associated interstitial lung disease and idiopathic pulmonary fibrosis: shared mechanistic and phenotypic traits suggest overlapping disease mechanisms. Rev Invest Clin 2015;67:280–6. 26696331 PMC4690466

[10] Roubille C, Haraoui B. Interstitial lung diseases induced or exacerbated by DMARDS and biologic agents in rheumatoid arthritis: a systematic literature review. Semin Arthritis Rheum 2014;43:613–26. 10.1016/j.semarthrit.2013.09.005 24231065

[11] Paul SK, Montvida O, Best JH, et al. Effectiveness of biologic and non-biologic antirheumatic drugs on anaemia markers in 153,788 patients with rheumatoid arthritis: new evidence from real-world data. Semin Arthritis Rheum 2018;47:478–84. 10.1016/j.semarthrit.2017.08.001 28947313

[12] Kerschbaumer A, Sepriano A, Smolen JS, et al. Efficacy of pharmacological treatment in rheumatoid arthritis: a systematic literature research informing the 2019 update of the EULAR recommendations for management of rheumatoid arthritis. Ann Rheum Dis 2020;79:744–59. 10.1136/annrheumdis-2019-216656 32033937 PMC7286044

[13] Guidelli GM, Viapiana O, Luciano N, et al. Efficacy and safety of baricitinib in 446 patients with rheumatoid arthritis: a real-life multicentre study. Clin Exp Rheumatol 2021;39:868–73. 10.55563/clinexprheumatol/pudtpo 33338001

[14] Antoniou KM, Mamoulaki M, Malagari K, et al. Infliximab therapy in pulmonary fibrosis associated with collagen vascular disease. Clin Exp Rheumatol 2007;25:23–8. 17417986

[15] Lindsay K, Melsom R, Jacob BK, et al. Acute progression of interstitial lung disease: a complication of etanercept particularly in the presence of rheumatoid lung and methotrexate treatment. Rheumatology (Oxford) 2006;45:1048–9. 10.1093/rheumatology/kel090 16760195

[16] Page MJ, McKenzie JE, Bossuyt PM, et al. The PRISMA 2020 statement: an updated guideline for reporting systematic reviews. BMJ 2021;372:n71. 10.1136/bmj.n71 33782057 PMC8005924

[17] Cumpston M, Li T, Page MJ, et al. Updated guidance for trusted systematic reviews: a new edition of the cochrane handbook for systematic reviews of interventions. Cochrane Database Syst Rev 2019;10:ED000142. 10.1002/14651858.ED000142 31643080 PMC10284251

[18] Hultcrantz M, Rind D, Akl EA, et al. The GRADE working group clarifies the construct of certainty of evidence. J Clin Epidemiol 2017;87:4–13. 10.1016/j.jclinepi.2017.05.006 28529184 PMC6542664

[19] Atienza-Mateo B, Remuzgo-Martínez S, Prieto-Peña D, et al. Rituximab in the treatment of interstitial lung disease associated with autoimmune diseases: experience from a single referral center and literature review. J Clin Med 2020;9:3070. 10.3390/jcm9103070 32977717 PMC7598697

[20] Fernández-Díaz C, Castañeda S, Melero-González RB, et al. Abatacept in interstitial lung disease associated with rheumatoid arthritis: national multicenter study of 263 patients. Rheumatology (Oxford) 2020;59:3906–16. 10.1093/rheumatology/keaa621 33068439

[21] Mena-Vázquez N, Redondo-Rodríguez R, Rojas-Gimenez M, et al. Efficacy and safety of rituximab in autoimmune disease-associated interstitial lung disease: a prospective cohort study. J Clin Med 2022;11:927. 10.3390/jcm11040927 35207203 PMC8879100

[22] Narváez J, Robles-Pérez A, Molina-Molina M, et al. Real-world clinical effectiveness of rituximab rescue therapy in patients with progressive rheumatoid arthritis-related interstitial lung disease. Semin Arthritis Rheum 2020;50:902–10. 10.1016/j.semarthrit.2020.08.008 32906025

[23] Cassone G, Manfredi A, Atzeni F, et al. Safety of abatacept in Italian patients with rheumatoid arthritis and interstitial lung disease: a multicenter retrospective study. J Clin Med 2020;9:277. 10.3390/jcm9010277 31963908 PMC7019755

[24] d’Alessandro M, Perillo F, Metella Refini R, et al. Efficacy of baricitinib in treating rheumatoid arthritis: modulatory effects on fibrotic and inflammatory biomarkers in a real-life setting. Int Immunopharmacol 2020;86:106748. 10.1016/j.intimp.2020.106748 32645631

[25] Fui A, Bergantini L, Selvi E, et al. Rituximab therapy in interstitial lung disease associated with rheumatoid arthritis. Intern Med J 2020;50:330–6. 10.1111/imj.14306 30963656

[26] Manfredi A, Cassone G, Furini F, et al. Tocilizumab therapy in rheumatoid arthritis with interstitial lung disease: a multicentre retrospective study. Intern Med J 2020;50:1085–90. 10.1111/imj.14670 31661185

[27] Tardella M, Di Carlo M, Carotti M, et al. A retrospective study of the efficacy of JAK inhibitors or abatacept on rheumatoid arthritis-interstitial lung disease. Inflammopharmacology 2022;30:705–12. 10.1007/s10787-022-00936-w 35462572 PMC9135879

[28] Detorakis EE, Magkanas E, Lasithiotaki I, et al. Evolution of imaging findings, laboratory and functional parameters in rheumatoid arthritis patients after one year of treatment with anti-TNF-alpha agents. Clin Exp Rheumatol 2017;35:43–52. 27908307

[29] Dixon WG, Hyrich KL, Watson KD, et al. Influence of anti-TNF therapy on mortality in patients with rheumatoid arthritis-associated interstitial lung disease: results from the British society for rheumatology biologics register. Ann Rheum Dis 2010;69:1086–91. 10.1136/ard.2009.120626 20444754 PMC2935328

[30] Druce KL, Iqbal K, Watson KD, et al. Mortality in patients with interstitial lung disease treated with rituximab or TNFi as a first biologic. RMD Open 2017;3:e000473. 10.1136/rmdopen-2017-000473 28955489 PMC5604605

[31] Duarte AC, Porter JC, Leandro MJ. The lung in a cohort of rheumatoid arthritis patients-an overview of different types of involvement and treatment. Rheumatology (Oxford) 2019;58:2031–8. 10.1093/rheumatology/kez177 31089697

[32] Md Yusof MY, Kabia A, Darby M, et al. Effect of rituximab on the progression of rheumatoid arthritis–related interstitial lung disease: 10 years’ experience at a single centre. Rheumatology (Oxford) 2017;56:1348–57. 10.1093/rheumatology/kex072 28444364 PMC5850796

[33] Koo BS, Hong S, Kim YJ, et al. Mortality in patients with rheumatoid arthritis-associated interstitial lung disease treated with an anti-tumor necrosis factor agent. Korean J Intern Med 2015;30:104–9. 10.3904/kjim.2015.30.1.104 25589842 PMC4293548

[34] Kurata I, Tsuboi H, Terasaki M, et al. Effect of biological disease-modifying anti-rheumatic drugs on airway and interstitial lung disease in patients with rheumatoid arthritis. Intern Med 2019;58:1703–12. 10.2169/internalmedicine.2226-18 30799358 PMC6630137

[35] Matteson EL, Bongartz T, Ryu JH, et al. Open-label, pilot study of the safety and clinical effects of rituximab in patients with rheumatoid arthritis-associated interstitial pneumonia. OJRA 2012;02:53–8. 10.4236/ojra.2012.23011

[36] Saldarriaga-Rivera LM, López-Villegas VJ. Janus kinase inhibitors as a therapeutic option in rheumatoid arthritis and associated interstitial lung disease. Revista Colombiana de Reumatología (English Edition) 2019;26:137–9. 10.1016/j.rcreue.2018.02.003

[37] Vacchi C, Manfredi A, Cassone G, et al. Tofacitinib for the treatment of severe interstitial lung disease related to rheumatoid arthritis. Case Reports in Medicine 2021;2021:1–5. 10.1155/2021/6652845 PMC8084679 33976699

[38] Solomon JJ, Chung JH, Cosgrove GP, et al. Predictors of mortality in rheumatoid arthritis-associated interstitial lung disease. Eur Respir J 2016;47:588–96. 10.1183/13993003.00357-2015 26585429

[39] Bongartz T, Nannini C, Medina-Velasquez YF, et al. Incidence and mortality of interstitial lung disease in rheumatoid arthritis: a population-based study. Arthritis & Rheumatism 2010;62:1583–91. 10.1002/art.27405 20155830 PMC4028137

[40] Brooks R, Baker JF, Yang Y, et al. The impact of disease severity measures on survival in U.S. veterans with rheumatoid arthritis-associated interstitial lung disease. Rheumatology (Oxford) 2022;61:4667–77. 10.1093/rheumatology/keac208 35377443 PMC9960484

[41] Kelly CA, Nisar M, Arthanari S, et al. Rheumatoid arthritis related interstitial lung disease - improving outcomes over 25 years: a large multicentre UK study. Rheumatology (Oxford) 2021;60:1882–90. 10.1093/rheumatology/keaa577 33150434

[42] Mochizuki T, Ikari K, Yano K, et al. Long-term deterioration of interstitial lung disease in patients with rheumatoid arthritis treated with abatacept. Mod Rheumatol 2019;29:413–7. 10.1080/14397595.2018.1481566 29798700

[43] Fernández-Díaz C, Atienza-Mateo B, Castañeda S, et al. Abatacept in monotherapy vs combined in interstitial lung disease of rheumatoid arthritis-multicentre study of 263 caucasian patients. Rheumatology (Oxford) 2021;61:299–308. 10.1093/rheumatology/keab317 33779697

[44] Xu J, Xiao L, Zhu J, et al. Methotrexate use reduces mortality risk in rheumatoid arthritis: a systematic review and meta-analysis of cohort studies. Semin Arthritis Rheum 2022;55:152031. 10.1016/j.semarthrit.2022.152031 35671648

[45] Fraenkel L, Bathon JM, England BR, et al. American college of rheumatology guideline for the treatment of rheumatoid arthritis. Arthritis Rheumatol 2021;73:1108–23. 10.1002/art.41752 34101376

[46] Kelly CA, Saravanan V, Nisar M, et al. Rheumatoid arthritis-related interstitial lung disease: associations, prognostic factors and physiological and radiological characteristics--a large multicentre UK study. Rheumatology (Oxford) 2014;53:1676–82. 10.1093/rheumatology/keu165 24758887

[47] Xie S, Li S, Chen B, et al. Serum anti-citrullinated protein antibodies and rheumatoid factor increase the risk of rheumatoid arthritis-related interstitial lung disease: a meta-analysis. Clin Rheumatol 2021;40:4533–43. 10.1007/s10067-021-05808-2 34189672

[48] Raghu G, Remy-Jardin M, Richeldi L, et al. Idiopathic pulmonary fibrosis (an update) and progressive pulmonary fibrosis in adults: an official ATS/ERS/JRS/ALAT clinical practice guideline. Am J Respir Crit Care Med 2022;205:e18–47. 10.1164/rccm.202202-0399ST 35486072 PMC9851481

