# Supplementary material for: Efficacy of non-conventional synthetic DMARDs for patients with rheumatoid arthritis-associated interstitial lung disease: a systematic review and meta-analysis
Source: RMD Open. 2023 Oct 29;9(4):e003487. doi: 10.1136/rmdopen-2023-003487 (PMC10619071; doi:10.1136/rmdopen-2023-003487)
Supplement: Supplementary data [file rmdopen-2023-003487supp001.pdf]

SUPPLEMENT MATERIAL

Supplement appendix 1

PUBMED:

#1 Rheumatoid Arthritis[MeSH Terms]
#2 Interstitial Lung Disease[MeSH Terms]
#3 Interstitial Pneumonia[Title/Abstract]
#4 Interstitial Pneumonitis[Title/Abstract]
#5 #2 OR #3 OR #4
#6 #1 AND #5
#7 Tumor Necrosis Factor-alpha[MeSH Terms]
#8 Etanercept[MeSH Terms]
#9 TNF Receptor Fusion Protein [Title/Abstract]
#10 Enbrel[Title/Abstract]
#11 Infliximab[MeSH Terms]
#12 Remicade[Title/Abstract]
#13 Adalimumab[MeSH Terms]
#14 Humira[Title/Abstract]
#15 D2E7 Antibody[Title/Abstract]
#16 Certolizumab[MeSH Terms]
#17 Cimzia[Title/Abstract]
#18 Golimumab[MeSH Terms]
#19 Simponi[Title/Abstract]
#20 Tocilizumab[MeSH Terms]
#21 Receptors, Interleukin-6[MeSH Terms]
#22 Monoclonal antibody[Title/Abstract]
#23 Actemra[Title/Abstract]
#24 Roactemra[Title/Abstract]
#25 Rituximab[MeSH Terms]
#26 CD20 Antibody, Rituximab [Title/Abstract]
#27 Mabthera[Title/Abstract]
#28 Rituxan[Title/Abstract]
#29 Abatacept[MeSH Terms]
#30 CTLA-4-Ig[Title/Abstract]
#31 Orencia[Title/Abstract]
#32 Janus kinase inhibitor[MeSH Terms]
#33 Janus kinase[MeSH Terms]
#34 Janus kinase 1 inhibitor[Title/Abstract]
#35 Janus kinase 2 inhibitor[Title/Abstract]
#36 Janus kinase 3 inhibitor[Title/Abstract]
#37 Tofacitinib[MeSH Terms]
#38 Xeljanz[Title/Abstract]
#39 CP-690550[Title/Abstract]

#40 Baricitinib[MeSH Terms]
#41 Olumiant[Title/Abstract]
#42 #7 OR #8 OR #9 OR #10 OR #11 OR #12 OR #13 OR #14 OR #15 OR #16 OR #17 OR #18 OR #19 OR #20 OR #21 OR #22 OR #23 OR #24 OR #25 OR #26 OR #27 OR #28 OR #29 OR #30 OR #31 OR #32 OR #33 OR #34 OR #35 OR #36 OR #37 OR #38 OR #39 OR #40 OR #41
#43 #6 AND #42
#44 Filters English
#45 Filters humans

EMBASE:

1 exp Rheumatoid Arthritis/
2 exp Interstitial Lung Disease/
3 Etanercept/
4 Etanercept.tw.
5 TNF Receptor Fusion Protein.tw.
6 Enbrel.tw.
7 Infliximab/
8 Infliximab.tw.
9 Remicade.tw.
10 Adalimumab/
11 Adalimumab.tw.
12 Humira.tw.
13 D2E7 Antibody.tw.
14 Certolizumab pegol/
15 Certolizumab.tw.
16 Cimzia.tw.
17 Golimumab/
18 Golimumab.tw.
19 Simponi.tw.
20 Tocilizumab/
21 Tocilizumab.tw.
22 Monoclonal antibody.tw.
23 Actemra.tw.
24 Roactemra.tw.
25 Rituximab/
26 Rituximab.tw.
27 CD20 Antibody, Rituximab.tw.
28 Mabthera.tw.
29 Rituxan.tw.
30 Abatacept/
31 Abatacept.tw.
32 CTLA-4-Ig.tw.
33 Orencia.tw.

34 Janus kinase inhibitor/
35 Janus kinase/
36 Janus kinase 1 inhibitor/
37 Janus kinase 2 inhibitor/
38 Janus kinase 3 inhibitor/
39 Tofacitinib/
40 Tofacitinib.tw.
41 Xeljanz.tw.
42 CP-690550.tw.
43 Baricitinib/
44 Baricitinib.tw.
45 Olumiant.tw.
46 INCB-028050.tw.
47 LY-3009104.tw
48 1 and 2
49 or/3-47
50 48 and 49
51 limit 50 to english language
52 limit 51 to human

the Cochrane Library

1 MeSH descriptor: [Arthritis, Rheumatoid] explode all trees
2 MeSH descriptor: [Lung Disease, Interstitial] explode all trees
3 MeSH descriptor: [Antibodies, Monoclonal] explode all trees
4 MeSH descriptor: [Monokines] explode all trees
5 MeSH descriptor: [Receptors, Interleukin-6] explode all trees
6 MeSH descriptor: [Tumor Necrosis Factor-alpha] explode all trees
7 MeSH descriptor: [Immunoglobulin g] explode all trees
8 MeSH descriptor: [Immunoconjugates] explode all trees
9 MeSH descriptor: [Polyethylene Glycols] explode all trees
10 MeSH descriptor: [Immunoglobulin Fab Fragments] explode all trees
11 MeSH descriptor: [T-Lymphocytes] explode all trees
12 MeSH descriptor: [Janus kinase] explode all trees
12 (Etanercept):ti,ab,kw
13 (TNF Receptor Fusion Protein):ti,ab,kw
14 (Enbrel.tw):ti,ab,kw
15 (Infliximab):ti,ab,kw
16 (Remicade):ti,ab,kw
17 (Adalimumab):ti,ab,kw
18 (Humira):ti,ab,kw
19 (D2E7 Antibody):ti,ab,kw
20 (Certolizumab):ti,ab,kw

21 (Cimzia):ti,ab,kw
22 (Golimumab):ti,ab,kw
23 (Simponi):ti,ab,kw
24 (Tocilizumab):ti,ab,kw
25 (Actemra):ti,ab,kw
26 (Roactemra):ti,ab,kw
27 (Rituximab):ti,ab,kw
28 (CD20 Antibody, Rituximab):ti,ab,kw
29 (Mabthera):ti,ab,kw
30 (Rituxan):ti,ab,kw
31 (Abatacept):ti,ab,kw
32 (CTLA-4):ti,ab,kw
33 (Orencia):ti,ab,kw
34 (Janus kinase inhibitor):ti,ab,kw
35 (Tofacitinib):ti,ab,kw
36 (Xeljanz):ti,ab,kw
37 (CP-690550):ti,ab,kw
38 (Baricitinib):ti,ab,kw
39 (Olumiant):ti,ab,kw
40 (INCB-028050):ti,ab,kw
41 (LY-3009104):ti,ab,kw
42 (#1 AND #2)
43 (#3 OR #4 OR #5 OR #6 OR #7 OR #8 OR #9 OR #10 OR #11 OR #12 OR #13 OR #14 OR #15 OR #16 OR #17 OR #18 OR #19 OR #20 OR #21 OR #22 OR #23 OR #24 OR #25 OR #26 OR #27 OR #28 OR #29 OR #30 OR #31 OR #32 OR #33 OR #34 OR #35 OR #36 OR #37 OR #38 OR #39 OR #40 OR #41)
44 (#42 AND #43)
45 (limit 44 to in trials)

Web of Science:
#1 TS=(Arthritis, Rheumatoid*)
#2 TS=(Lung Disease, Interstitial*)
#3 TS=(Interstitial Pneumonia*)
#4 TS=(Interstitial Pneumonitis*)
#5 #2 OR #3 OR #4 ((TS=(Lung Disease, Interstitial*)) OR TS=(Interstitial Pneumonia*)) OR TS=(Interstitial Pneumonitis*)
#6 #1 AND #5
#7 TS=(Etanercept*)
#8 TS=(TNF Receptor Fusion Protein*)
#9 TS=(Enbrel*)
#10 TS=(Infliximab*)
#11 TS=(Remicade*)
#12 TS=(Adalimumab*)

#13 TS=(Humira*)
#14 TS=(D2E7 Antibody*)
#15 TS=(Certolizumab*)
#16 TS=(Cimzia*)
#17 TS=(Golimumab*)
#18 TS=(Simponi*)
#19 TS=(Tocilizumab*)
#20 TS=(Monoclonal antibody*)
#21 TS=(Actemra*)
#22 TS=(Roactemra*)
#23 TS=(Rituximab*)
#24 TS=(CD20 Antibody, Rituximab*)
#25 TS=(Mabthera*)
#26 TS=(Rituxan*)
#27 TS=(Abatacept*)
#28 TS=(CTLA-4*)
#29 TS=(Orencia*)
#30 TS=(Janus kinase inhibitor*)
#33 TS=(Janus kinase*)
#37 TS=(Tofacitinib*)
#38 TS=(Xeljanz*)
#39 TS=(CP-690550*)
#40 TS=(Baricitinib*)
#41 TS=(Olumiant*)
#42 #7 OR #8 OR #9 OR #10 OR #11 OR #12 OR #13 OR #14 OR #15 OR #16 OR #17 OR #18 OR #19 OR #20 OR #21 OR #22 OR #23 OR #24 OR #25 OR #26 OR #27 OR #28 OR #29 OR #30 OR #31 OR #32 OR #33 OR #34 OR #35 OR #36 OR #37 OR #38 OR #39 OR #40 OR #41
#43 #6 AND #42
#44 #6 AND #42 **and** English (Languages)
#45 #6 AND #42 **and** English (Languages) **and** Humans (MeSH Headings)

Supplement Table 1. Details of the Newcastle-Ottawa Scale in all included studies

Study	Selection				Comparability	0utcomes			Scores
	Representativeness of the exposed cohort	Selection of the non-exposed cohort	Ascertainment of exposure	Demonstration that outcome of interest was not present at start of study	control for important factor	Assessment of outcomes	Follow-up long enough for outcomes to occur*	Adequacy of follow up of cohorts†	
Atienza-Mateo 2020[1]	0	0	1	1	1	1	0	1	5
Cassone 2020[2]	1	1	1	1	1	1	1	1	8
d'Alessandro 2020[3]	1	1	1	1	1	0	0	1	6
Detorakis 2016[4]	1	1	1	1	2	1	0	1	8
Dixon 2010[5]	1	1	1	1	1	1	1	1	8
Druce 2017[6]	1	1	1	1	1	1	1	1	8
Duarte 2019[7]	1	1	1	1	1	1	1	0	7
Fernández-Diaz 2020[8]	1	1	1	1	2	1	1	0	8
Fui 2019[9]	1	1	1	1	1	1	0	1	7
Koo 2015[10]	1	1	1	1	1	0	1	1	7
Kurata 2019[11]	1	1	1	1	2	1	0	1	8
Manfredi 2019[12]	1	1	1	1	1	1	1	1	8
Matteson 2012[13]	1	1	1	1	2	1	0	0	7
Mena-Vázquez 2022[14]	1	1	1	1	2	1	1	1	9
Narváez 2020[15]	1	1	1	1	1	1	1	0	7
Tardella 2022[16]	1	1	1	1	1	1	0	1	7
Yusof 2017[17]	1	1	1	1	1	0	1	0	6

* If the follow-up time have been more than 20 months, the follow-up was considered as long enough.

† If the number of patients >80% at the end of follow-up, the cohort was considered to be adequacy.

References

1. Atienza-Mateo B, Remuzgo-Martinez S, Prieto-Pena D, et al. Rituximab in the Treatment of Interstitial Lung Disease Associated with Autoimmune Diseases: Experience from a Single Referral Center and Literature Review. *J Clin Med* 2020;9(10) doi: 10.3390/jcm9103070 [published Online First: 2020/09/27]

2. Cassone G, Manfredi A, Atzeni F, et al. Safety of Abatacept in Italian Patients with Rheumatoid Arthritis and Interstitial Lung Disease: A Multicenter Retrospective Study. *J Clin Med* 2020;9(1) doi: 10.3390/jcm9010277 [published Online First: 2020/01/23]

3. d'Alessandro M, Perillo F, Metella Refini R, et al. Efficacy of baricitinib in treating rheumatoid arthritis: Modulatory effects on fibrotic and inflammatory biomarkers in a real-life setting. *Int Immunopharmacol* 2020;86:106748. doi: 10.1016/j.intimp.2020.106748 [published Online First: 2020/07/10]

4. Detorakis EE, Magkanas E, Lasithiotaki I, et al. Evolution of imaging findings, laboratory and functional parameters in rheumatoid arthritis patients after one year of treatment with anti-TNF-alpha agents. *Clin Exp Rheumatol* 2017;35(1):43-52.

5. Dixon WG, Hyrich KL, Watson KD, et al. Influence of anti-TNF therapy on mortality in patients with rheumatoid arthritis-associated interstitial lung disease: results from the British Society for Rheumatology Biologics Register. *Ann Rheum Dis* 2010;69(6):1086-91. doi: 10.1136/ard.2009.120626 [published Online First: 2010/05/07]

6. Druce KL, Iqbal K, Watson KD, et al. Mortality in patients with interstitial lung disease treated with rituximab or TNFi as a first biologic. *RMD Open* 2017;3(1):e000473. doi: 10.1136/rmdopen-2017-000473 [published Online First: 2017/09/29]

7. Duarte AC, Porter JC, Leandro MJ. The lung in a cohort of rheumatoid arthritis patients-an overview of different types of involvement and treatment. *Rheumatology (Oxford)* 2019;58(11):2031-38. doi: 10.1093/rheumatology/kez177 [published Online First: 2019/05/16]

8. Fernandez-Diaz C, Castaneda S, Melero-Gonzalez RB, et al. Abatacept in interstitial lung disease associated with rheumatoid arthritis: national multicenter study of 263 patients. *Rheumatology (Oxford)* 2020;59(12):3906-16. doi: 10.1093/rheumatology/keaa621 [published Online First: 2020/10/18]

9. Fui A, Bergantini L, Selvi E, et al. Rituximab therapy in interstitial lung disease associated with rheumatoid arthritis. *Intern Med J* 2020;50(3):330-36. doi: 10.1111/imj.14306 [published Online First: 2019/04/10]

10. Koo BS, Hong S, Kim YJ, et al. Mortality in patients with rheumatoid arthritis-associated interstitial lung disease treated with an anti-tumor necrosis factor agent. *Korean J Intern Med* 2015;30(1):104-9. doi: 10.3904/kjim.2015.30.1.104 [published Online First: 2015/01/16]

11. Kurata I, Tsuboi H, Terasaki M, et al. Effect of Biological Disease-modifying Anti-rheumatic Drugs on Airway and Interstitial Lung Disease in Patients with Rheumatoid Arthritis. *Intern Med* 2019;58(12):1703-12. doi: 10.2169/internalmedicine.2226-18 [published Online First: 2019/02/26]

12. Manfredi A, Cassone G, Furini F, et al. Tocilizumab therapy in rheumatoid arthritis with interstitial lung disease: a multicentre retrospective study. *Intern Med J* 2020;50(9):1085-90. doi: 10.1111/imj.14670 [published Online First: 2019/10/30]

13. Matteson EL, Bongartz T, Ryu JH, et al. Open-Label, Pilot Study of the Safety and Clinical Effects of Rituximab in Patients with Rheumatoid Arthritis-Associated Interstitial Pneumonia. *Open J Rheumatol Autoimmune Dis* 2012;02(03):53-58. doi: 10.4236/ojra.2012.23011

14. Mena-Vazquez N, Redondo-Rodriguez R, Rojas-Gimenez M, et al. Efficacy and Safety of Rituximab in Autoimmune Disease-Associated Interstitial Lung Disease: A Prospective Cohort Study. *J Clin Med* 2022;11(4) doi: 10.3390/jcm11040927 [published Online First: 2022/02/26]

15. Narvaez J, Robles-Perez A, Molina-Molina M, et al. Real-world clinical effectiveness of rituximab rescue therapy in patients with progressive rheumatoid arthritis-related interstitial lung disease. *Semin Arthritis Rheum* 2020;50(5):902-10. doi: 10.1016/j.semarthrit.2020.08.008 [published Online First: 2020/09/10]

16. Tardella M, Di Carlo M, Carotti M, et al. A retrospective study of the efficacy of JAK inhibitors or abatacept on rheumatoid arthritis-interstitial lung disease. *Inflammopharmacology* 2022;30(3):705-12. doi: 10.1007/s10787-022-00936-w [published Online First: 2022/04/26]

17. Md Yusof MY, Kabia A, Darby M, et al. Effect of rituximab on the progression of rheumatoid arthritis–related interstitial lung disease: 10 years' experience at a single centre. *Rheumatology* 2017;56(8):1348-57. doi: 10.1093/rheumatology/kex072

Supplement Table 2. Summary of findings and certainty of evidence for efficacy

	Summary of findings		Certainty of evidence				Certainty of evidence
	No of participants (No of trials)	Mean difference (95% CI)	Study design	Inconsistency	Imprecision	Small study effects	
**FVC**							
ABA	243 (3)	0.37 (-2.29 to 3.03)	Downgraded*	Not downgraded	Not downgraded	Not downgraded	Moderate
RTX	124 (7)	-4.62 (-8.90 to -0.33)	Downgraded*	Not downgraded	Not downgraded	Not downgraded	Moderate
TCZ	25 (1)	3.00 (-7.89 to 13.89)	Downgraded*	Not downgraded	Downgraded†	Downgraded‡	Very low
TNFis	42 (1)	-0.36 (-8.70 to 7.98)	Downgraded*	Not downgraded	Not downgraded	Downgraded‡	Low
JAKis	31 (1)	-1.59 (-6.84 to 3.66)	Downgraded*	Not downgraded	Not downgraded	Downgraded‡	Low
**DLCO**							
ABA	177 (2)	-0.65 (-3.58 to 2.28)	Downgraded*	Not downgraded	Not downgraded	Not downgraded	Moderate
RTX	77 (4)	0.01 (-4.16 to 4.17)	Downgraded*	Not downgraded	Not downgraded	Not downgraded	Moderate
TCZ	25 (1)	1.50 (-9.83 to 12.83)	Downgraded*	Not downgraded	Downgraded†	Downgraded‡	Very low
JAKis	31 (1)	-3.03 (-8.17 to 2.11)	Downgraded*	Not downgraded	Not downgraded	Downgraded‡	Low
**FEV1**	75 (3)	0.16 (-5.85 to 6.17)	Downgraded*	Not downgraded	Not downgraded	Not downgraded	Moderate
**Non-progression rate of HRCT**							
ABA	216 (3)	0.804 (0.747 to 0.856)	Downgraded*	Not downgraded	Not downgraded	Not downgraded	Moderate
RTX	102 (5)	0.661 (0.542 to 0.773)	Downgraded*	Not downgraded	Not downgraded	Not downgraded	Moderate
TCZ	28 (1)	0.929 (0.765 to 0.991)	Downgraded*	Not downgraded	Not downgraded	Downgraded‡	Low
JAKis	31 (1)	0.839 (0.663 to 0.945)	Downgraded*	Not downgraded	Not downgraded	Downgraded‡	Low
NA	23 (1)	0.696 (0.471 to 0.868)	Downgraded*	Not downgraded	Not downgraded	Downgraded‡	Low
**Case fatality rate**							
ABA	263(1)	0.008 (0.001 to 0.027)	Downgraded*	Not downgraded	Not downgraded	Downgraded‡	Low
RTX	117 (3)	0.165 (0.100 to 0.240)	Downgraded*	Not downgraded	Not downgraded	Not downgraded	Moderate
TNFis	632 (3)	0.064 (0.045 to 0.085)	Downgraded*	Not downgraded	Not downgraded	Not downgraded	Moderate

FVC, forced vital capacity; DLCO, diffusion lung capacity for carbon monoxide; FEV1, forced expiratory volume in the first second; HRCT, high-resolution computed tomography; ABA, abatacept; RTX, rituximab; TCZ, tocilizumab; TNFis, tumor necrosis factor inhibitors; JAKis, Janus kinase inhibitors

* Downgraded by one level because studies were cohort studies with a one-level reduction in evidence level

† Downgraded by one level because the limits of the 95% confidence interval were 20 points different to smallest worthwhile effect

‡ Downgraded by one level owing to small study bias
